# Involvement of HO-1 and Autophagy in the Protective Effect of Magnolol in Hepatic Steatosis-Induced NLRP3 Inflammasome Activation In Vivo and In Vitro

**DOI:** 10.3390/antiox9100924

**Published:** 2020-09-27

**Authors:** Ni-Chun Kuo, Shieh-Yang Huang, Chien-Yi Yang, Hsin-Hsueh Shen, Yen-Mei Lee

**Affiliations:** 1 School of Medicine, National Defense Medical Center, Taipei 11490, Taiwan; as123as41as@gmail.com; 2 Department of Medical Education, Taichung Veterans General Hospital, Taichung 40705, Taiwan; 3 Department of Pharmacy, Kaohsiung Armed Forces General Hospital, Kaohsiung 80284, Taiwan; keflex33@gmail.com; 4 Division of General Surgery, Department of Surgery, Tri-Service General Hospital Sungshan Branch, Taipei 10581, Taiwan; wayneyoung680324@gmail.com; 5 Department and Graduate Institute of Pharmacology, National Defense Medical Center, Taipei 11490, Taiwan; 6 Department of Pharmacy Practice, Tri-Service General Hospital, National Defense Medical Center, Taipei 11490, Taiwan

**Keywords:** magnolol, HO-1 (heme oxygenase-1), autophagy, hyperglycemia, nonalcoholic fatty liver disease (NAFLD)

## Abstract

Magnolol (MG) is the main active compound of *Magnolia officinalis* and exerts a wide range of biological activities. In this study, we investigated the effects of MG using tyloxapol (Tylo)-induced (200 mg/kg, i.p.) hyperlipidemia in rats and palmitic acid (PA)-stimulated (0.3 mM) HepG2 cells. Our results showed that Tylo injection significantly increased plasma levels of triglyceride and cholesterol as well as superoxide anion in the livers, whereas MG pretreatment reversed these changes. MG reduced hepatic lipogenesis by attenuating sterol regulatory element-binding protein-1c (SREBP-1c) and fatty acid synthase (FAS) proteins and *Srebp-1*, *Fas*, *Acc*, and *Cd36* mRNA expression as well as upregulated the lipolysis-associated genes *Hsl*, *Mgl*, and *Atgl*. Furthermore, MG reduced plasma interleukin-1β (IL-1β) and protein expression of NLR family pyrin domain-containing 3 (NLRP3), apoptosis-associated speck-like protein (ASC), and caspase 1 as well as upregulated nuclear translocation of nuclear factor erythroid 2-related factor 2 (Nrf2) and induction of heme oxygenase-1 (HO-1) in hepatocytes of Tylo-treated rats. Enhanced autophagic flux by elevation of autophagy related protein 5-12 (ATG5-12), ATG7, Beclin1, and microtubule-associated protein light chain 3 B II (LC3BII)/LC3BI ratio, and reduction of sequestosome-1 (SQSTM1/p62) and phosphorylation of mTOR was observed by MG administration. However, autophagy inhibition with 3-methyladenine (3-MA) in HepG2 cells drastically abrogated the MG-mediated suppression of inflammation and lipid metabolism. In conclusion, MG inhibited hepatic steatosis-induced NLRP3 inflammasome activation through the restoration of autophagy to promote HO-1 signaling capable of ameliorating oxidative stress and inflammatory responses.

## 1. Introduction

Nonalcoholic fatty liver disease (NAFLD) is the leading cause of liver disease worldwide, with the prevalence ranging from 25% to 45% [1] and characterized by excessive lipid accumulation in the hepatocytes. The pathogenesis includes a broad spectrum of hepatic changes such as steatosis, steatohepatitis, and cirrhosis [2]. Excess free fatty acid, inflammatory disorders, and oxidative stress are the main risk factors in the pathological changes of NAFLD [3,4]. Although many medications have been investigated to address the therapeutic potentials of NAFLD, no effective treatment has so far been approved. Thus, the development of new therapeutic strategies capable of improving the pathogenesis of NAFLD warrants urgent exploration.

Nuclear factor erythroid 2-related factor 2 (Nrf2) regulates antioxidant machinery and expresses a variety of antioxidant genes, including heme oxygenase-1 (HO-1) and superoxide dismutase 2 (SOD2), which alleviate oxidative stress and inflammatory responses in liver diseases [5,6]. NLR family pyrin domain-containing 3 (NLRP3) inflammasome is a multiprotein complex consisting of NLRP3, apoptosis-associated speck-like protein (ASC), and the effector protein procaspase-1. Upon activation, the assembly of NLRP3 inflammasome components results in the activation of caspase 1 and further triggers the proteolytic cleavage of prointerleukin-1β (proIL-1β) and prointerleukin-18 (proIL-18) into mature IL-1β and IL-18, respectively [7,8]. These cytokines subsequently precipitate inflammatory cascades and play a vital role in the development of NAFLD [9]. Overt activation of NLRP3 inflammasome exacerbated hepatic steatosis, whereas defective inflammasome activation exhibited significant improvement of hepatocyte steatosis, inflammation, and fibrogenesis [10]. Recent investigation also suggests that the upregulation of HO-1 confers protective effects against fulminant hepatic failure by inhibition of NLRP3 inflammasome activation [11].

Autophagy is a lysosomal degradative process that transports organelles and proteins to lysosome for degradation, and this pathway promotes cell survival by supplying energy under stress conditions [12]. Defective hepatic autophagy is a common feature of NAFLD progression, which is accompanied by hepatic lipid metabolism, endoplasmic reticulum stress, and insulin resistance [13,14]. Furthermore, autophagy is involved in attenuation of NLRP3 inflammasome and liver injury [15]. Consequently, therapies aimed at restoring the autophagic flux might improve the pathological progress of NAFLD.

An emerging interest has been focused on the bioactive compounds that mitigate the progression of NAFLD. Polyphenols, such as thymoquinone [16,17], resveratrol [18], and pomegranate [19], have been reported to increase antioxidant capacities and HO-1 expression to regulate hepatic steatosis and inflammation [20]. Magnolol (MG) is the main bioactive compound derived from *Magnolia officinalis*, which has been shown to exert anti-inflammation, anti-oxidation, and hepatoprotective properties [21,22]. MG reduced inflammation through inhibition of nuclear factor-kappa B (NF-κB) activation in TNF-α-stimulated human aortic endothelial cells [23]. In addition, MG improved fasting glucose and plasma insulin in type 2 diabetic Goto-Kakizaki rats without changing the body weight [24] and enhanced glucose uptake in 3T3-L1 cells [25]. However, its underlying mechanism in the protective effects of NAFLD remains obscure. This study aimed to investigate the effects of MG in tyloxapol (Tylo)-injected rats and palmitic acid (PA)-induced hepatic steatosis in HepG2 cells and whether Nrf2/HO-1 signaling and autophagy are involved in the protective effects of MG.

## 2. Materials and Methods

### 2.1. Animal Experimentation

All procedures for animal handling were conducted in accordance with the Guide for the Care and Use of Laboratory Animals published by the US National Institutes of Health (NIH Publication No. 85-23, revised in 1996). Male Wistar rats (8 weeks old, 300–350 g) were obtained from BioLASCO Co., Ltd., Taipei, Taiwan. This study was approved by the Institutional Animal Care and Use Committee of the National Defense Medical Center, Taipei, Taiwan (IACUC-19-306). Hypertriglyceridemia was induced by a single injection of tyloxapol (Tylo; 200 mg/kg, i.p.), as previously described [26]. Rats were randomly divided into the following three groups: (1) Ctrl group: (*n* = 5); (2) Tylo group: rats were fasted overnight for 8 h and received an injection with Tylo (200 mg/kg, i.p.) (Carbosynth, Compton, Berkshire, UK) (*n* = 12); and (3) MG+Tylo group: rats were cotreated with magnolol (10 mg/kg, i.p.) (Sigma-Aldrich, St. Louis, MO, USA) and Tylo injection (*n* = 9). After Tylo administration for 18 h, rats were then sacrificed and blood and liver samples were collected for further analysis.

### 2.2. Cell Culture and Palmitate-Induced Steatosis

Human hepatoma cell line HepG2 was obtained from American Type Culture Collection (ATCC, Manassas, VA, USA) and cultured in Dulbecco’s Modified Eagle’s Medium (DMEM, Gibco Life Technologies, Grand Island, NY, USA) containing 10% fetal bovine serum at 37 °C in a humidified atmosphere containing 5% CO_2_. Cellular steatosis was induced by treatment with 0.3 mM palmitic acid (PA) (Sigma-Aldrich, St. Louis, MO, USA) conjugated with serum-free bovine albumin for 24 h. HepG2 cells were divided into the following four groups (*n* = 5 for each group): (1) Ctrl group: cells were treated with 0.1% DMSO; (2) MG group: cells were treated with magnolol (8 μg/mL); (3) PA group: cells were treated with 0.3 mM palmitic acid for 24 h; and (4) PA+MG group: cells were pretreated with magnolol (8 μg/mL) for 6 h before PA exposure.

### 2.3. Cell Viability of MG

The cell viability was measured by the 3-(4,5-dimethylthiazol-2-yl)-2,5-diphenyltetrazolium bromide assay. Briefly, 10^4^ cells/well were placed into 96-well plates and treated with progressive doses of magnolol (0, 2, 4, 8, 16, and 32 μg/mL) for 24 h. Cell viability was determined by colorimetry (Thermo Fisher Scientific, Pittsburgh, PA, USA) using MTT assay. Insoluble formazan crystals were dissolved in dimethyl sulfoxide (DMSO) and measured at 490 nm, according to the manufacturer’s instructions.

### 2.4. Oil Red O Staining

After treatment with PA, the culture medium was discarded, and cells were soaked with paraformaldehyde (4%) at room temperature for 30 min and washed with PBS. After staining with Oil Red O solution, cell culture dishes were immersed in 60% isopropanol and detected by a light microscope (Nikon, Melville, NY, USA). Oil droplets were eluted by administration with isopropanol and optical density was measured using a spectrophotometer at 520 nm (BioTek, Winooski, VT, USA).

### 2.5. Measurement of Triglyceride, Total Cholesterol, and IL-1β Levels in Tylo-Injected Rats and PA-Stimulated HepG2 Cells

Plasma levels of triglyceride (TG) and total cholesterol (TCHO) in the rats were detected by a Fuji DRI-CHEM 3030 analyzer (Fuji Photo Film, Tokyo, Japan). Plasma and cellular IL-1β levels (Cloud-Clone Corp., Houston, TX, USA) and cellular triglyceride levels (BioVision, Milpitas, CA, USA) of the supernatant in HepG2 cells were measured using enzyme-linked immunosorbent assay (ELISA) kit according to the manufacturer’s instructions.

### 2.6. Detection of Superoxide Formation in the Liver of Tylo-injected Rats and Reactive Oxygen Species Formation in PA-Stimulated HepG2 Cells

Superoxide anion production was determined by lucigenin-derived chemiluminescence. Briefly, samples were placed into a 96-well plate filled with 200 μL modified Krebs-HEPES solution and placed in a microplate luminometer (Hidex, Microplate Luminometer, Turku, Finland). After recording background counts, the sample was added to each well and incubated with lucigenin (125 μM) for 1 min. Counts were then recorded for each well, and the respective background was subtracted. After recording, the liver was dried in a drying cabinet for 24 h. These results were expressed as counts per second (cps) per milligram dry weight of tissues.

Reactive oxygen species (ROS) of HepG2 cells was measured by DHE (Dihydroethidium) Assay Kit (Abcam, Cambridge, MA, USA) according to the manufacturer’s protocol. DHE was used as a fluorescent probe for the detection of ROS generation and specific for superoxide and hydrogen peroxide formation. ROS generation is represented as total DHE fluorescence at 510 nm on a microplate reader (Thermo Fisher Scientific, Pittsburgh, PA, USA).

### 2.7. Western Blot Analysis

Liver samples were homogenized and centrifuged using RIPA lysis buffer (Millipore, Bedford, MA, USA) containing a protease inhibitor cocktail with 1 mmol/L phenylmethanesulfonyl fluoride. Protein lysates were quantified using the BCA kit (Thermo Scientific, Waltham, MA, USA). Twenty micrograms of protein extract were separated on 10% sodium dodecyl sulfate–polyacrylamide gels and transferred to a nitrocellulose membrane. After blocking in 5% bovine serum albumin for 1 h, membranes were then incubated overnight at 4 °C with the following primary antibodies: LC3, ATG7, p62/SQSTM1, mTOR, p-mTOR, ULK, Beclin1 ATG5-12, SOD2, β-actin, NLRP3, ASC, caspase 1, HSL, ATGL, and FAS (all 1 : 2000, Cell Signaling Technology, Danvers, MA, USA); SREBP-1c (1 : 2000, Santa Cruz Biotechnology, Santa Cruz, CA, USA); IL-6 (1:1,000, Novus Biologicals, Centennial, CO, USA); HO-1 ( 1: 2000, Enzo Life Sciences, Farmingdale, NY, USA); and TNF-α (1 : 1000, Abcam, Cambridge, MA, USA) at 4 °C overnight. After washing with TBST, the membranes were incubated with horseradish peroxidase-conjugated IgG secondary antibodies (1:3000, Cell Signaling). The density of the individual protein bands was quantified by densitometric scanning of the blots using ImageJ software (version 1.50d, USA). Expression levels were then normalized to the ctrl group, which were set to 1.

### 2.8. RNA Isolation and Quantitative Reverse Transcription-Polymerase Chain Reaction (qRT-PCR)

Total RNA was extracted using Trizol reagent (Invitrogen, Carlsbad, CA, USA) according to the manufacturer’s protocol. Reverse transcription was carried out using the High Capacity cDNA Reverse Transcription Kit (Applied Biosystems^TM^, Waltham, MA, USA) and using a 20 μL reaction volume containing 0.5–1 μg RNA with 150 ng random primers, 10 μM dNTPs, and RNase Inhibitor. PCR reactions were performed in 10 μL volumes using PowerUp SYBR Green Master Mix (Applied Biosystems^TM^) with each primer at a concentration of 0.05 μM. The specific primer sequences are shown in Table 1.

### 2.9. Statistical Analysis

Data were plotted using GraphPad PRISM software (8.4.0, CA, USA) and are presented as the mean ± SEM. Statistical analysis was performed with one-way analysis of variance (ANOVA) followed by the Newman–Keuls post hoc multiple comparison methods. *p*-values < 0.05 were considered statistically significant.

## 3. Results

### 3.1. MG Alleviated Hyperlipidemia and ROS Formation in the Liver of Tylo-Injected Rats

**Figure 1:**
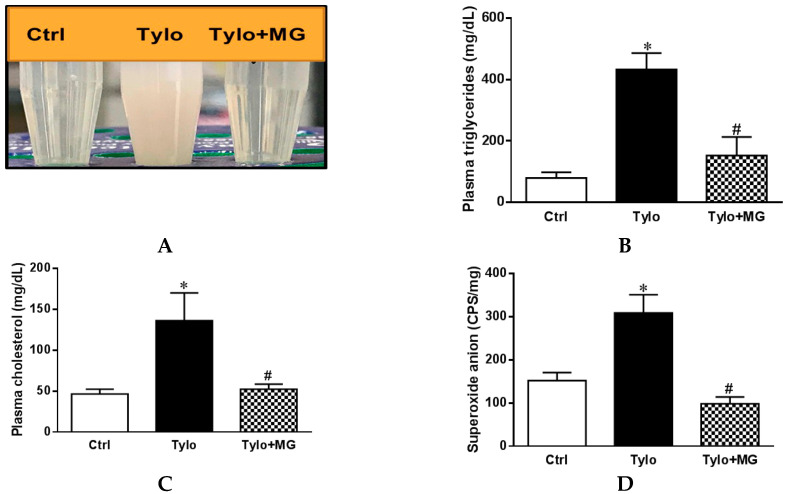
Effects of magnolol (MG) treatment on plasma lipid profiles and hepatic superoxide anion in tyloxapol (Tylo)-injected rats. (**A**) Plasma levels of triglyceride (TG); (**B**) macroscopic photograph of plasma; (**C**) plasma levels of cholesterol (TC); (**D**) superoxide anion formation. Data are expressed as mean ± SEM, *n* = 5–12. * *p* < 0.05 vs. Ctrl; # *p* < 0.05 vs. Tylo (tyloxapol).

Excessive accumulation of triglycerides results in hyperlipidemia and plays an important role in the development of NAFLD [27]. Tyloxapol, a nonionic surfactant that blocks plasma lipolytic functions, is widely implicated in establishing the experimental model of acute hyperlipidemia-associated NAFLD [28,29]. As shown in Figure 1A–C, administration with Tylo in rats displayed obvious suspension and a significant increase in the plasma levels of triglycerides (TGs) and cholesterol (TC) as compared to the Ctrl group (*p* < 0.05). In contrast, MG treatment significantly decreased Tylo-provoked hyperlipidemia in rats. In addition, Tylo injection resulted in a remarkable increase in superoxide anion formation as compared with the Ctrl group (309.0 ± 42.3 vs. 152.2 ± 18.7 cps/mg tissue) (*p* < 0.05) (Figure 1D). Treatment of Tylo-injected rats with MG drastically reduced hepatic superoxide anion formation (98.6 ± 15.6 cps/mg tissue). These findings indicated that MG improved hyperlipidemia and possessed antioxidant capacity in Tylo-injected rats.

### 3.2. Effect of MG Treatment on Lipogenesis and Lipolysis Profiles in the Liver of Tylo-induced Hyperlipidemia in Rats

**Figure 2:**
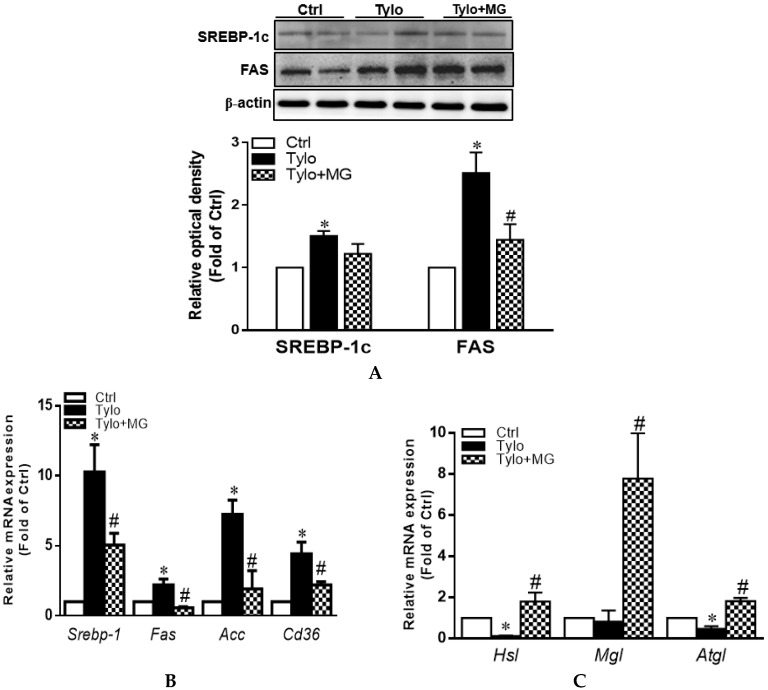
Effects of magnolol (MG) treatment on lipid homeostasis in the liver of tyloxapol-injected rats. (**A**) Representative Western blot and densitometry analysis of sterol regulatory element-binding protein-1c (SREBP-1c) and fatty acid synthase (FAS); (**B**) mRNA expression of *Srebp1*, *Fas*, *Acc*, and *Cd36*; (**C**) mRNA expression of *Hsl*, *Mgl*, and *Atgl*. Data are expressed as mean ± SEM, *n* = 5–12. * *p* < 0.05 vs. Ctrl; ^#^ *p* < 0.05 vs. Tylo (tyloxapol).

Lipid accumulation in hepatocytes is the primary stage of the progression of hepatic steatosis. Sterol regulatory element-binding protein-1c (SREBP-1c) is the major transcription factor in the regulation of lipogenic de novo biosynthesis by activating acetyl-CoA carboxylase (ACC) and fatty acid synthase (FAS) in the liver [30]. To examine the role of MG in the regulation of lipid homeostasis, we evaluated protein and gene expression involving lipogenesis and lipolysis. As shown in Figure 2A, protein expression of SREBP-1c and FAS were significantly upregulated by Tylo injection, while MG administration exhibited lower levels of SREBP-1c and FAS protein expression (*p* < 0.05). In addition, the mRNA levels of *Srebp-1*, *Fas*, *Acc*, and *Cd36* were significantly elevated in the liver of Tylo-injected rats, which were all diminished by treatment with MG (*p* < 0.05) (Figure 2B). Figure 2C shows that significant induction of lipolysis genes *Hsl*, *Mgl*, and *Atgl* was observed after MG treatment (Figure 2C). These results suggested MG preserved hepatic lipid homeostasis by modulating lipogenesis and lipolysis.

### 3.3. MG Attenuated Proinflammatory Cytokines and NLRP3 Inflammasome Activation in Tyloxapol-Induced Hyperlipidemia in Rats

**Figure 3:**
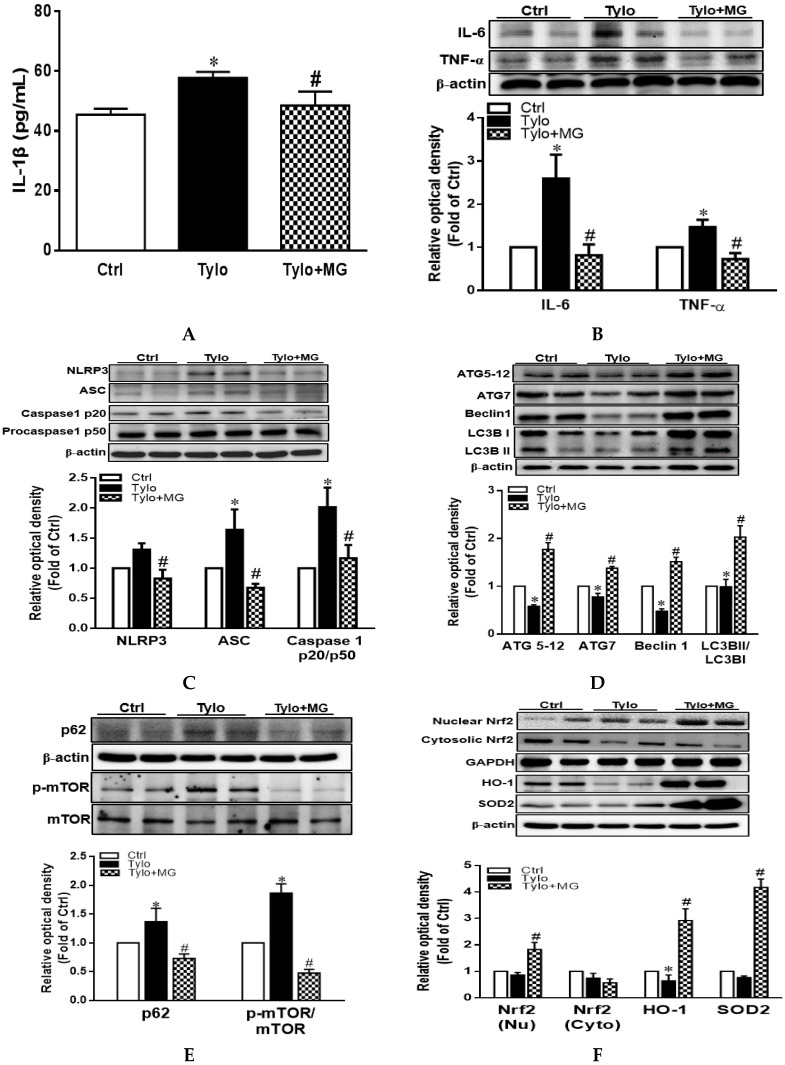
Effects of magnolol (MG) treatment on interleukin-1β (IL-1β) secretion and NLR family pyrin domain-containing 3 (NLRP3) inflammasome activation in the liver of tyloxapol-injected rats. (**A**) Plasma levels of IL-1β; (**B**) representative Western blot and densitometry analysis of tumor necrosis factor-α (TNF-α) and interleukin-6 (IL-6); (**C**) representative Western blot and densitometry analysis of NLRP3, apoptosis-associated speck-like protein (ASC), and caspase 1 p20 and p50; (**D**) representative Western blot and densitometry analysis of autophagy related protein 5-12 (ATG5-12), ATG7, Beclin1, and microtubule-associated protein light chain 3 B II (LC3BII)/LC3BI ratio (**E**) representative Western blot and densitometry analysis of sequestosome-1 (SQSTM1/p62) and phosphorylation of mTOR; (**F**) representative Western blot and densitometry analysis of nuclear/cytosolic Nrf2 and the downstream HO-1 and SOD2. Data are expressed as mean ± SEM, *n* = 5–12. * *p* < 0.05 vs. Ctrl; ^#^ *p* < 0.05 vs. Tylo (tyloxapol).

IL-1β is an essential inflammatory mediator involving in NAFLD pathogenesis, which is regulated by NLRP3 inflammasome [31]. Plasma IL-1β displayed significantly higher levels in the Tylo group than those of the Ctrl group, while MG treatment drastically reversed the increase in plasma IL-1β levels (Figure 3A). Moreover, protein expression of TNF-α and IL-6 was significantly elevated in the Tylo-treated group, an effect that was normalized by MG administration (Figure 3B). It is well established that the maturation of the proinflammatory cytokine IL-1β is elicited by the proteolytic cleavage of caspase 1, which is mediated by the activation of NLRP3 inflammasome [32]. Results in Figure 3C show that protein expression of NLRP3, apoptosis-associated speck-like protein containing a CARD (ASC), and caspase1 p20/p50 ratio were significantly increased by Tylo injection as compared to Ctrl group (*p* < 0.05). Treatment with MG exhibited considerably lower levels of NLRP3, caspase1 p20/p50 ratio, and ASC than those of the Tylo group (Figure 3C).

### 3.4. MG Induced Hepatic Autophagy Activation and Nrf2/HO-1 Signaling in Tyloxapol-Induced Hyperlipidemia in Rats

The two ubiquitinated substrates conjugate ATG 5-12 and LC3B, which are recognized as essential markers for the detection of autophagosome formation [33]. To explore whether the decreased hepatic activation of the NLRP3 inflammasome of MG was mediated via the activation of autophagy, we next evaluated the expression of autophagy-associated proteins in the liver of Tylo-injected rats. Figure 3D shows that protein expression of ATG 5-12, ATG7, Beclin1, and LC3B II/LC3B I ratio were significantly lower in Tylo-injected rats than those of Ctrl rats. Administration with MG displayed substantially higher levels of ATG 5-12, ATG7, Beclin1, and LC3B II/LC3B I ratio. Furthermore, p62/SQSTM1 serves as cargo adaptors for the degradation of ubiquitinated substrates by lysosome and its expression inversely correlates with autophagic degradation [34]. The results in Figure 3E show that p62/SQSTM1 and phosphorylation of mTOR were markedly enhanced in Tylo-injected rats, an effect that was significantly reversed by MG treatment (*p* < 0.05), indicative of MG in the restoration of autophagy.

Nrf2 is the major endogenous antioxidant machinery capable of the induction of HO-1 for oxidative stress alleviation [35]. Western blot analysis showed MG administration significantly upregulated protein expression of nuclear Nrf2 and its downstream HO-1 and SOD2 as compared to the Tylo group (*p* < 0.05). These results indicated that MG exerted the beneficial effects via the induction of antioxidant Nrf2/HO-1 signaling.

### 3.5. Cell Viability of HepG2 Cells Treated with MG

**Figure 4:**
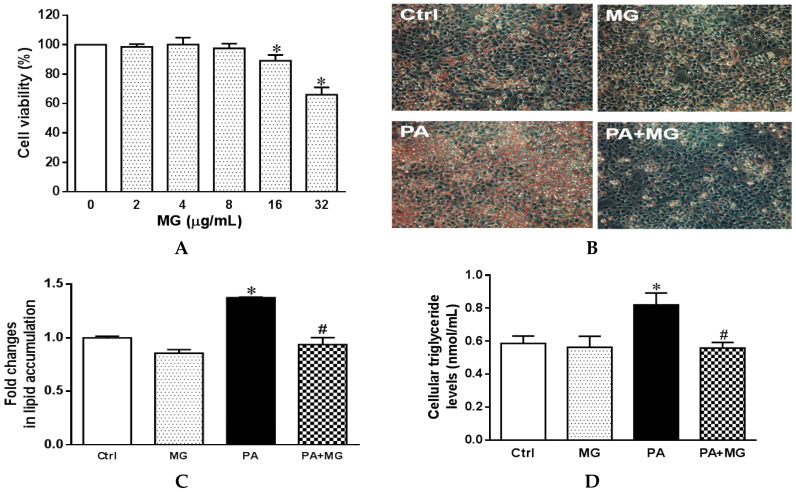
Effects of magnolol (MG) treatment on cell viability and lipid accumulation in steatotic HepG2 cells. (**A**) Cell viability of HepG2 cells treated with MG (0–32 μM) for 24 h expressed as optical density percentage; (**B**) photomicrograph of Oil Red O staining (20×); (**C**) colorimetric assay for quantification; (**D**) triglyceride levels of the supernatant fraction in HepG2 cells. Data are expressed as mean ± SEM, *n* = 4. * *p* < 0.05 vs. Ctrl; ^#^ *p* < 0.05 vs. palmitic acid (PA).

Lipotoxicity is generally induced by incubation with saturated fatty acids in vitro, such as palmitic acid (16:0), the most abundant saturated fatty acid in the diet and circulation [36]. To evaluate the cell viability of MG, HepG2 cells were treated with progressive doses of MG (0, 2, 4, 8, 16, and 32 µg/mL) for 24 h. The effect on MG on cell viability was assessed by MTT assay. The results showed that no significant toxicity was observed at MG concentrations up to 8 µg/mL (Figure 4A). Therefore, an amount of 8 µg/mL of MG was employed for further analysis.

### 3.6. MG Alleviated Steatosis in PA-Stimulated Hepatocytes

For the PA-induced steatosis cell model, the cells were treated with 0.3 mM PA conjugated with serum-free bovine albumin for 24 h. Oil Red O stain showed PA treatment exhibited a marked increase in lipid accumulation in comparison to the Ctrl group, an effect that was significantly normalized by MG treatment (Figure 4B,C). In addition, triglyceride levels were significantly upregulated after PA stimulation, whereas the addition of MG to steatotic HepG2 cells significantly reduced the TG contents (Figure 4D).

### 3.7. MG Regulated Lipid Homeostasis in PA-Stimulated HepG2 Cells

**Figure 5:**
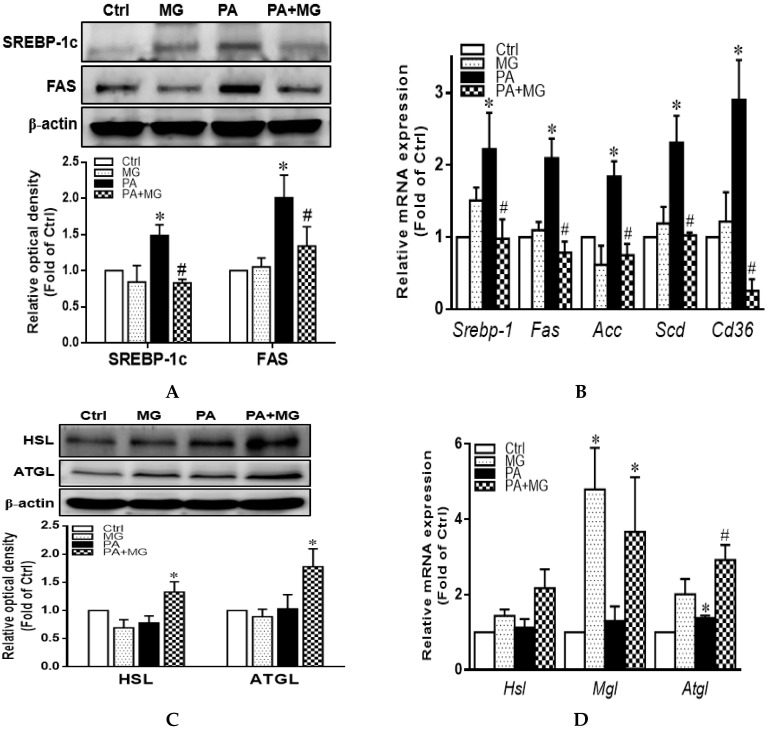
Effects of magnolol (MG) treatment on lipid homeostasis in steatotic HepG2 cells. (**A**) Representative Western blot and densitometry analysis of SREBP-1c and FAS; (**B**) relative mRNA expression of lipogenesis markers *Srebp-1*, *Fas*, *Acc*, *Scd*, and Cd36; (**C**) representative Western blot and densitometry analysis of HSL and ATGL; (**D**) relative mRNA expression of lipolysis markers *Hsl*, *Mgl*, and *Atgl*. Data are expressed as mean ± SEM, *n* = 4. * *p* < 0.05 vs. Ctrl; ^#^ *p* < 0.05 vs. PA (palmitic acid).

Protein expression of SREBP-1c and FAS as well as mRNA levels of *Srebp-1*, *Fas*, *Acc*, *Scd*, and *Cd36* were significantly increased in the presence of PA (*p* < 0.05) (Figure 5A,B). MG treatment drastically reversed these increases (Figure 5A,B). Furthermore, protein expression of lipolysis markers HSL and ATGL as well as mRNA levels of *Hsl*, *Mgl*, and *Atgl* were notably increased by MG treatment (Figure 5C,D). These results suggested that MG preserved lipid homeostasis with decreased lipogenesis and enhanced lipolysis in PA-induced steatotic HepG2 cells.

### 3.8. MG Attenuated IL-1β Secretion and NLRP3 Inflammasome Activation in PA-Induced Steatotic HepG2 Cells

**Figure 6:**
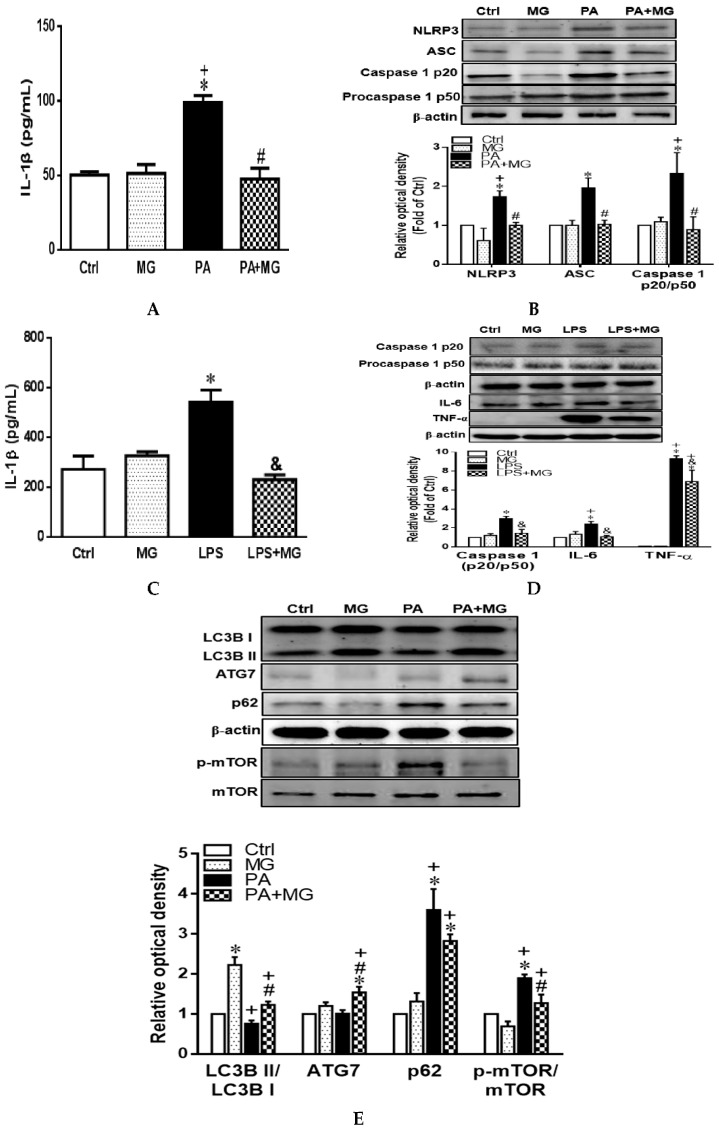
Magnolol (MG) inhibited the activation of NLRP3 inflammasome in PA-induced steatosis and lipopolysaccharide (LPS)-challenged HepG2 cells. (**A**) IL-1β protein levels in the supernatant fraction of steatotic cells; (**B**) representative Western blot and densitometry analysis of NLRP3, ASC, caspase1 p20, and procaspase1 p50 in steatotic cells; (**C**) IL-1β protein levels in the supernatant fraction of LPS-challenged cells; (**D**) representative Western blot and densitometry analysis of caspase1 p20, procaspase1 p50, IL-6, and TNF-α in LPS-challenged cells; (**E**) representative Western blot and densitometry analysis of autophagy-related markers LC3B, ATG7, p62/SQSTM1, and mTOR in steatotic cells. Data are expressed as mean ± SEM, *n* = 4. * *p* < 0.05 vs. Ctrl; ^+^ *p* < 0.05 vs. MG; ^#^ *p* < 0.05 vs. PA; ^&^ *p* < 0.05 vs. LPS.

Figure 6A shows that incubation of HepG2 cells with PA resulted in the significant upregulation of IL-1β secretion as compared to the Ctrl group (*p* < 0.05), whereas MG treatment significantly reversed the increase of IL-1β. Western blot revealed that NLRP3, ASC expression, and caspase 1 p20/p50 ratio were increased dramatically in PA-treated hepatocytes, an effect that was normalized by MG administration (Figure 6B).

### 3.9. MG Attenuated NLRP3 Inflammasome and Proinflammatory Cytokine in LPS-Stimulated HepG2 Cells

As a consequence of increased intestinal permeability and intestinal bacterial overgrowth in patients with NAFLD, plasma lipopolysaccharide (LPS) is elevated and mediates inflammatory cascade in the liver [37]. Thus, LPS-challenged HepG2 cells were implicated in mimicking inflammatory responses of NAFLD in vitro. HepG2 cells were incubated with LPS (100 ng/mL) for 18 h and post-treated with MG (8 µg/mL) for 6 h. Figure 6C,D shows that incubation of HepG2 cells with LPS resulted in the significant upregulation of IL-1β secretion and protein expression of caspase 1 p20/p50 ratio, IL-6, and TNF-α as compared to the Ctrl group (*p* < 0.05), whereas MG treatment significantly reversed these increases.

### 3.10. Activation of Autophagy Contributed to the Protective Effects of MG against PA-Induced Lipotoxicity in Hepatocytes

Compared with the Ctrl group, PA-treated HepG2 cells exhibited significantly impaired autophagy activity evidenced by decreased LC3B II/LC3B I ratio and increased p62/SQSTM1 and phosphorylation of mTOR. However, MG markedly increased LC3B II/LC3B I ratio and ATG7, as well as reduced p62/SQSTM1 and p-mTOR expression, indicating MG possessed the capacity to reverse PA-induced impairment of autophagy flux (Figure 6E).

### 3.11. MG-Induced Inhibition of Lipid Accumulation, Triglyceride, and NLRP3 Inflammasome and Induction of HO-1 Depend on Autophagy Induction

**Figure 7:**
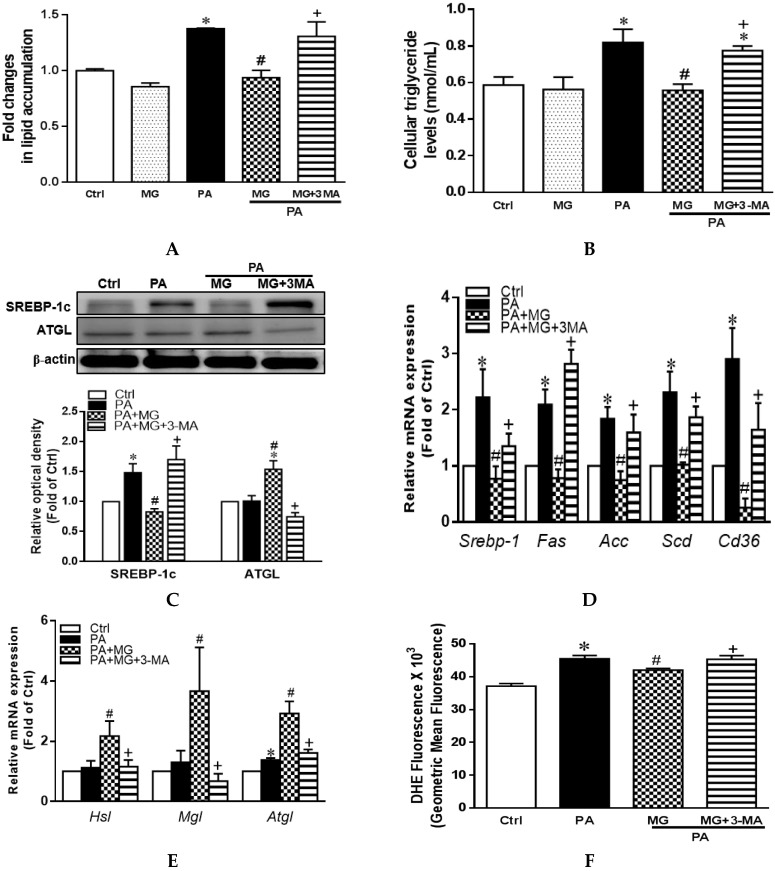

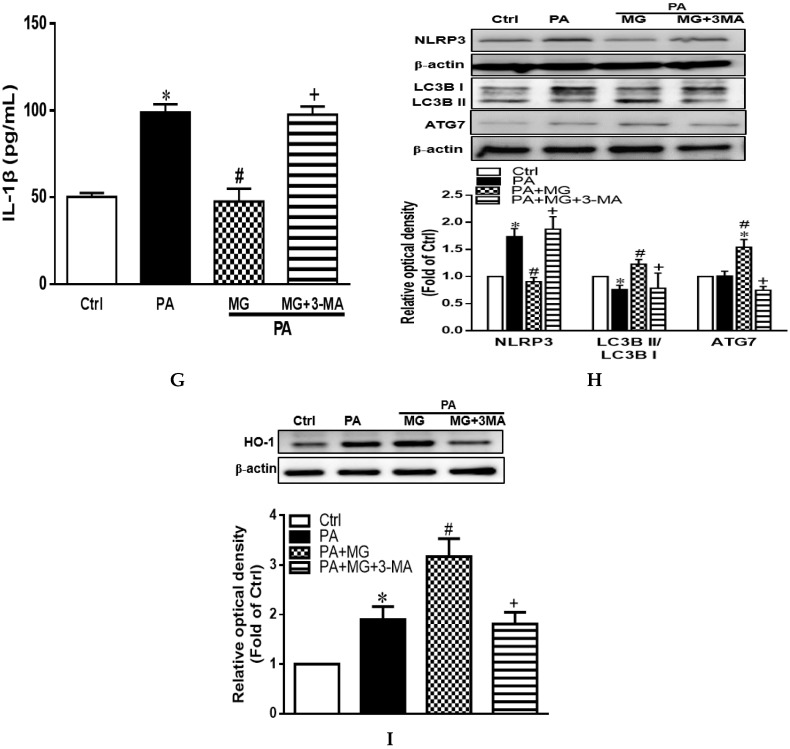
Effects of autophagy inhibitor 3-methyladenine (3-MA) in PA-induced steatosis of HepG2 cells. Cells were treated with MG in the presence or absence of 3-MA (5 mM) for 6 h, followed by treatment with PA (0.3 mM) for another 24 h. (**A**) Colorimetric assay for Oil Red O quantification; (**B**) triglyceride levels of the supernatant fraction; (**C**) representative Western blot and densitometry analysis of SREBP-1c and ATGL; (**D**) relative mRNA expression of lipogenesis markers *Srebp-1*, *Fas*, *Acc*, *Scd*, and *Cd36*; (**E**) relative mRNA expression of lipolysis markers Hsl, Mgl, and Atgl; (**F**) reactive oxygen species were measured by dihydroethidium (DHE) Assay; (**G**) IL-1β protein levels in the supernatant fraction; (**H**) representative Western blot and densitometry analysis of NLRP3, LC3B, and ATG7; (**I**) representative Western blot and densitometry analysis of HO-1. Data are expressed as mean ± SEM, *n* = 4. * *p* < 0.05 vs. Ctrl; ^#^ *p* < 0.05 vs. PA (palmitic acid); ^+^ *p* < 0.05 vs. MG (magnolol).

To confirm the hypothesis that MG reduced lipid deposition and the progression of NAFLD by activation of autophagy, we treated hepatocytes with autophagy inhibitor 3-methyladenine (3-MA, 5 mM) (Taiclone, Taipei, Taiwan, China) prior to MG exposure. Interestingly, pretreatment with 3-MA displayed a significant increase in lipid accumulation and triglyceride levels that were reduced by MG (Figure 7A,B). In addition, the effects of MG on protein expression of SREBP-1c and ATGL and gene expression of lipogenesis indicators *Srebp-1*, *Fas*, *ACC*, *Scd*, and *Cd36* as well as lipolysis *Hsl*, *Mgl*, and *Atgl* were all dramatically diminished by 3-MA administration (Figure 7C–E).

As expected, 3-MA treatment increased ROS formation (Figure 7F), IL-1β levels (Figure 7G), and NLRP3 protein expression as well as diminished the MG-induced increase of LC3B and ATG 7 (Figure 7H). In particular, the combined administration of 3-MA attenuated MG-mediated HO-1 expression (Figure 7I). Collectively, these results suggested that MG reduced intracellular lipid deposition by modulating various genes/proteins involved in lipid homeostasis via activation of autophagy to induce HO-1, thereby protecting against hyperlipidemia and NAFLD.

## 4. Discussion

NAFLD is characterized by the accumulation of lipid droplets in hepatocytes and hyperlipidemia, which results in systemic inflammatory responses and metabolic abnormalities. We showed that MG modulated lipid homeostasis by attenuating lipogenesis and increasing lipolysis in the liver of Tylo-injected rats and hepatocytes. MG induced Nrf2/HO-1 signaling, diminished Tylo-induced hyperlipidemia, oxidative stress, IL-1β as well as TNF-α and IL-6 that might arise from the suppression of NLRP3 inflammasome. Additionally, MG activated autophagic flux through the upregulation of ATG5-12, ATG7, Beclin1, and LC3BII/LC3BI ratio and reduction of p62/SQSTM1 and p-mTOR. However, autophagy inhibition in the presence of 3-MA dramatically abolished the beneficial effects of MG in lipid metabolism, activation of NLRP3 inflammasome, and suppressed HO-1 induction. These data suggested that MG induced Nrf1/HO-1 signaling through the activation of autophagy to ameliorate the pathogenesis of hepatic steatosis to NAFLD. Our results hence provide a novel insight that MG might be a promising strategy for NAFLD management.

Magnolol (5,5′-diallyl-2,2′-dihydroxybiphenyl) is a polyphenolic binaphthalene compound and a structural isomer of honokiol, both of which exert multifunctional activities. MG is predominantly distributed in the liver and the main metabolites, isomagnolol and tetrahydromagnolol, have been reported to exert synergistic potentials with MG or other molecules acting on cannabinoid receptors [38]. Emerging evidence supports that oxidative stress is mechanistically involved in the PA-induced cell death and pathogenesis of NAFLD [39]. Excessive free fatty acids and lipid accumulation in the liver result in lipotoxicity, leading to mitochondrial dysfunction and the production of reactive oxygen species (ROS) [40]. Oxidative stress and inflammatory responses play an essential role in the pathogenesis of NAFLD and metabolic diseases [3,41]. Therefore, it is conceivable that the induction of cellular antioxidant systems may provide beneficial effects against NAFLD. As an adaptive response to counteract oxidative stress, Nrf2 serves as a critical regulator of the antioxidant defense mechanism, which translocated to the nucleus and induces the target gene HO-1 expression through an antioxidant response element (ARE) [42]. We showed that MG administration increased the expression of HO-1 and SOD2 by promoting the translocation of Nrf2 into the nucleus. The induction of HO-1 and SOD2 led to the alleviation of oxidative stress and conferred resistance to inflammatory insults [6,43]. HO-1 is the rate-limiting enzyme of heme catabolism, which catalyzes the cellular heme to carbon monoxide (CO), biliverdin, and free iron and exerts significant anti-inflammatory effects [44]. Specifically, MG-stimulated Nrf2/HO-1 signaling is mediated through the activation of autophagy since pretreatment with 3-MA significantly reversed the effects of MG on HO-1 expression. This finding is consistent with previous literature that MG inhibited Gram-negative bacterium *Porphyromonas gingivalis* LPS-induced periodontitis and inflammation in macrophages via the activation of Nrf2/HO-1 signaling [45].

Dysregulated lipid metabolism results in lipotoxicity and induces oxidative stress and hepatic inflammation that might contribute to the progression of NAFLD [46]. Our results showed that MG decreased plasma TG and cholesterol and lipid accumulation in hepatocytes, indicating an essential role of MG in regulating lipid homeostasis. Moreover, SREBP-1c is a major transcription factor regulating lipogenesis and its downstream targets FAS, ACC, and SCD, and its activation aggravates the symptoms of NAFLD [47]. In the present study, MG treatment inhibited SREBP-1c activation and a subsequent reduction in fatty acid synthesis in Tylo-injected rats and steatotic HepG2 cells. This finding is consistent with recent literature that MG exerted protective effects against hepatic steatosis and hyperlipidemia through inhibition of SREBP-1c [22]. Consequently, our results further revealed that MG regulated SREBP-1c and metabolic reprogramming through the activation of autophagy.

The activation of NLRP3 inflammasome in response to cellular oxidative stress or pathogen-associated molecular patterns (PAMPs) provides essential signaling for the pathogenesis of NAFLD [48]. PA can be integrated by scavenger receptor CD36, which serves as a Toll-like receptor activator and endocytosis receptor, priming the activation of NLRP3 inflammasome [49]. Genetic ablation of NLRP3 prevented obesity-induced inflammasome and caspase 1 activation in the liver as well as improved insulin sensitivity [50]. Furthermore, pharmacological manipulation of the anti-NLRP3 inflammasome assembly ameliorated lipotoxicity [51]. Previous studies have elucidated that autophagic proteins negatively regulate the activation of NLRP3 inflammasome by preserving mitochondrial integrity [52]. In addition, NLRP3 inhibitor dampens liver inflammation in NAFLD progression and reverses liver scarring [31,53]. Our results showed that MG reduced NLRP3 inflammasome activation and ROS generation and augmented autophagy in the liver and hepatocytes. Notably, the effects were dramatically reversed by 3MA treatment, suggesting that the magnolol-mediated beneficial effects are autophagy-dependent.

Autophagy is a catabolic process of stress-induced cellular damage by lysosomal degradation and provides a fundamental mechanism to maintain cellular energetic balance. Accumulating evidence prompted the observation that autophagy was downregulated in the liver from patients with NAFLD [54]. Impaired hepatic autophagy prevented the clearance of the excess lipid, protein aggregates, and dysfunctional mitochondria, which might result in the development of hepatic steatosis [55]. The induction of autophagy confers protective effects against palmitic acid-induced lipotoxicity in hepatocytes [14]. Herein, MG increased ATG5-12, ATG7, Beclin1, ULK1, and LC3-II/I ratio and decreased p62/SQSTM1 and p-mTOR in the liver of Tylo-injected rats and PA-stimulated HepG2 cells, suggesting that MG might promote the formation of autophagic flux to alleviate hepatic steatosis. In addition, phosphorylation of p62 increases its affinity binding to Kelch-like ECH-associated protein 1 (Keap1), a Nrf2 inhibitor protein, and interferes with the Keap1–Nrf2 complex, resulting in the release and cytosolic stabilization of Nrf2 [56]. This process further induces Nrf2-ARE pathway activation. In response to the stressed condition, the low energy state results in the catabolism of p62/Keap1 and other autophagy substrates and thus triggers Nrf2 stabilization and nuclear translocation. In this study, MG activated autophagy flux and increased the degradation of p62/Keap1, leading to the nuclear translocation of Nrf2 and the induction of HO-1. Our results clearly showed that the protective effect of MG against lipotoxicity was entirely abolished by autophagy inhibition, indicating the close interplay between autophagy and HO-1.

Lipophagy is described as a selective autophagy process to degrade intracellular lipid droplets through autophagosome formation in hepatocytes [57]. It is established that p62/SQSTM1 is essential for LC3B recruitment to lipid droplets in ethanol-induced lipophagy, while knockdown of p62/SQSTM1 triggers the accumulation of triglyceride and cholesterol [58]. Recent investigations suggest that serum p62/SQSTM1 levels could be defined as a potential biomarker in the diagnosis of patients with steatosis and lobular inflammation [59]. Our results showed that MG attenuated p62/SQSTM1, accompanied by improvement of lipid accumulation, indicating MG reduced hepatic lipid accumulation associated with autophagosome degradation via p62/SQSTM1-involved lipophagy.

While our data are suggestive of the role for MG in alleviation of Tylo-induced NAFLD, the following limitations must be addressed. First, Tylo injection is an acute model to mimic NAFLD, which induces hyperlipidemia and hepatic lipid accumulation within a few hours and this is in contrast to the slow progression of NAFLD and does not reproduce the human metabolic syndrome. All metabolic changes and signal transduction that were investigated in this acute experiment reflect short-term alterations. Second, we did not explore the therapeutic effect of MG, which may confine if MG reverses established NAFLD, although our data only indicated the preventive effects.

## 5. Conclusions

**Figure 8:**
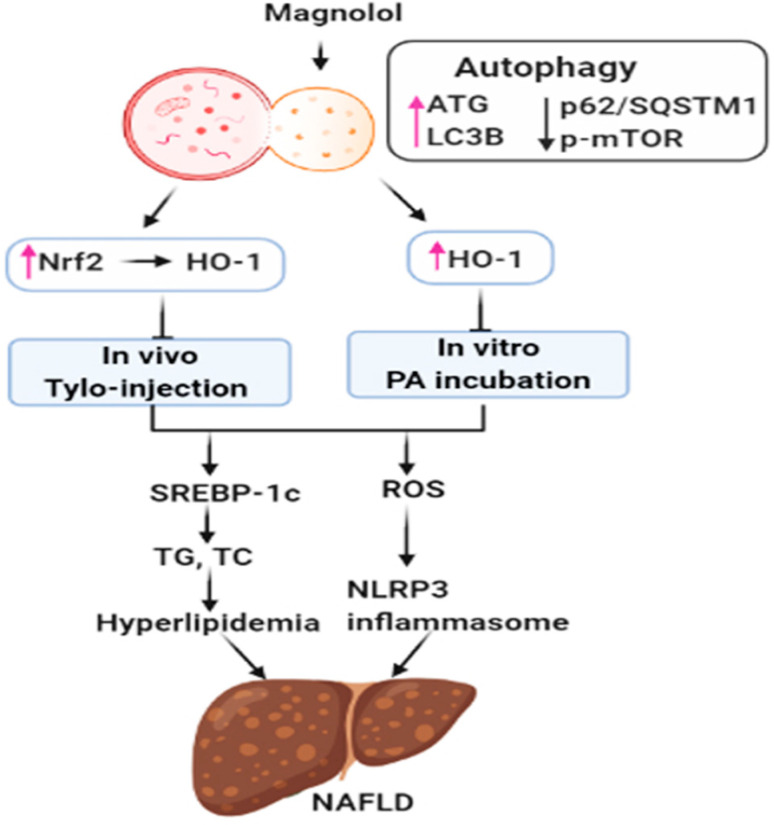
Schematic depiction of the protective mechanisms of magnolol (MG) in tyloxapol- and palmitic acid-induced NAFLD. MG activates autophagy by upregulation of ATG5-12, ATG7, Beclin1, and LC3B and reduction of p62/SQSTM1 and p-mTOR expression, which results in the activation of antioxidant Nrf2/HO-1 signaling, thereby suppressing tyloxapol and palmitic acid (PA)-induced hepatic steatosis and inflammation. In addition, MG inhibits hyperlipidemia through downregulation of SREBP-1c, as well as ROS and NLRP3 inflammasome activation.

In conclusion, as shown in Figure 8, our present study revealed that MG effectively reversed hepatic steatosis by modulating lipid accumulation, reducing NLRP3 inflammasome activation, and increasing cellular Nrf2/HO-1 antioxidant capacities. Activation of autophagy may contribute to the protective effects of MG. These findings provide a novel insight into the implication of MG in the management of NAFLD.

## Figures and Tables

**Table 1:** Real-time PCR primer sequences.

Gene	Forward (5′→3′)	Reverse (3′→5′)	Accession No.
*Fas*	AAGGACCTGTCTAGGTTTGATGC	TGGCTTCATAGGTGACTTCCA	NM_017332.1
*Srebp-1*	GCCCCTGTAACGACCACTG	CAGCGAGTCTGCCTTGATG	NM_001276707.1
*Acc*	ATGTCTGGCTTGCACCTAGTA	CCCCAAAGCGAGTAACAAATTCT	NM_022193.1
*Scd*	TCTAGCTCCTATACCACCACCA	TCGTCTCCAACTTATCTCCTCC	NM_139192.2
*Cd36*	GGCTGTGACCGGAACTGTG	AGGTCTCCAACTGGCATTAGAA	NM_031561.2
*Hsl*	TCAGTGTCTAGGTCAGACTGG	AGGCTTCTGTTGGGTATTGGA	NM_012859.1
*Mgl*	ATGCCAGAGGAAAGTTCCCC	CGTCTGCATTGACCAGGTG	NM_138502.2
*Atgl*	GGCTTCCTCGGCGTCTACTA	TTTACCAGGTTGAAGGAGGGG	NM_001108509.2
*β-actin*	AGGCACCAGGGCGTGAT	GCCCACATAGGAATCCTTCTGAC	NM_031144.3
