# Correction: Kuo et al. Involvement of HO-1 and Autophagy in the Protective Effect of Magnolol in Hepatic Steatosis-Induced NLRP3 Inflammasome Activation In Vivo and In Vitro. *Antioxidants* 2020, *9*, 924

**DOI:** 10.3390/antiox14060690

**Published:** 2025-06-05

**Authors:** Ni-Chun Kuo, Shieh-Yang Huang, Chien-Yi Yang, Hsin-Hsueh Shen, Yen-Mei Lee

**Affiliations:** 1 School of Medicine, National Defense Medical Center, Taipei 11490, Taiwan; as123as41as@gmail.com; 2 Department of Medical Education, Taichung Veterans General Hospital, Taichung 40705, Taiwan; 3 Department of Pharmacy, Kaohsiung Armed Forces General Hospital, Kaohsiung 80284, Taiwan; keflex33@gmail.com; 4 Division of General Surgery, Department of Surgery, Tri-Service General Hospital Sungshan Branch, Taipei 10581, Taiwan; wayneyoung680324@gmail.com; 5 Department and Graduate Institute of Pharmacology, National Defense Medical Center, Taipei 11490, Taiwan; 6 Department of Pharmacy Practice, Tri-Service General Hospital, National Defense Medical Center, Taipei 11490, Taiwan

In the original publication [1], there was a mistake in Figure 6E as published. The corrected Figure 6 appears below.

The authors state that the scientific conclusions are unaffected. This correction was approved by the Academic Editor. The original publication has also been updated.

## Figures and Tables

**Figure 6:**
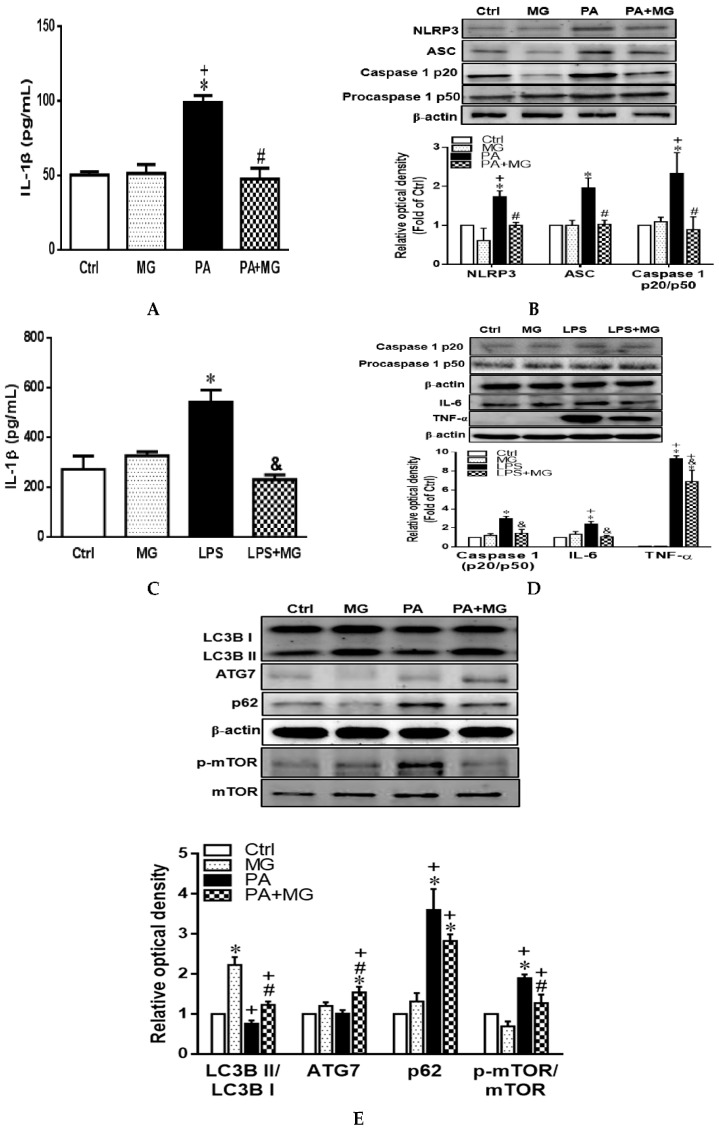
Magnolol (MG) inhibited the activation of NLRP3 inflammasome in PA-induced steatosis and lipopolysaccharide (LPS)-challenged HepG2 cells. (**A**) IL-1β protein levels in the supernatant fraction of steatotic cells; (**B**) representative Western blot and densitometry analysis of NLRP3, ASC, caspase1 p20, and procaspase1 p50 in steatotic cells; (**C**) IL-1β protein levels in the supernatant fraction of LPS-challenged cells; (**D**) representative Western blot and densitometry analysis of caspase1 p20, procaspase1 p50, IL-6, and TNF-α in LPS-challenged cells; (**E**) representative Western blot and densitometry analysis of autophagy-related markers LC3B, ATG7, p62/SQSTM1, and mTOR in steatotic cells. Data are expressed as mean ± SEM, *n* = 4. * *p* < 0.05 vs. Ctrl; ^+^ *p* < 0.05 vs. MG; ^#^ *p* < 0.05 vs. PA; ^&^ *p* < 0.05 vs. LPS.
